# High level of IgG4 as a biomarker for a new subset of inflammatory bowel disease

**DOI:** 10.1038/s41598-018-28397-8

**Published:** 2018-07-03

**Authors:** Zhujun Wang, Min Zhu, Chengxin Luo, Yu zhen, Jingxi Mu, Wenyan Zhang, Qin Ouyang, Hu Zhang

**Affiliations:** 1 Department of Gastroenterology, West China Hospital, Sichuan University, Chengdu, Sichuan China; 2 Department of Pathology, West China Hospital, Sichuan University, Chengdu, Sichuan China

## Abstract

The roles of B and plasma cells in the pathogenesis of inflammatory bowel disease (IBD) are largely unrevealed. Data on the characteristics of IgG4 in patients with IBD are scarce. In this case-control study, serum IgG4 levels were comparable between patients with IBD and healthy individuals, whereas patients with IBD had dramatically higher mucosal IgG4 counts than healthy individuals. In patients with UC, mucosal IgG4 counts were positively correlated with serum IgG4 levels, serum IgG4/IgG ratios, and the Mayo Index; serum IgG4 levels and IgG4/IgG ratios were associated with a history of intestinal surgery and medications. A significant mucosal IgG4 count was found in 33.3% of patients with IBD, whereas, elevated serum IgG4 levels were found in only 9.9% of patients with IBD. Lesions were more severe and extensive in IBD patients with high levels of serum and mucosal IgG4. High levels of serum and mucosal IgG4 decreased after treatment with glucocorticoids or other immunosuppressants. High IgG4 level may be a biomarker for a new subset of IBD. More studies are warranted to explore this new subset of IBD for personalized therapy in the future.

## Introduction

Inflammatory bowel disease (IBD), comprising ulcerative colitis (UC) and Crohn’s disease (CD), is a series of nonspecific inflammatory conditions affecting the gastrointestinal tract. Some multicenter studies and meta-analyses have demonstrated an increasing prevalence of IBD in developing countries, including China^1–3^. Although the pathogenesis of IBD has not been fully elucidated, it is acknowledged that genetic susceptibility, immune dysregulation, gut microbiota, and environmental triggers are strongly involved. Given the dysfunction of the intestinal barrier in the susceptible population, various antigens and pathogens in the lumen of the gut could continuously induce an immune response leading to a disturbance of intestinal immune homeostasis, characterized by continuous or remittent inflammation. Immune lymphocytes are mainly classified as T cells and B cells. In the past 20 years, most studies regarding the pathogenesis of IBD have been focused on T cells. A huge amount of evidence has confirmed that T lymphocytes are a critical subset of immune cells in the pathogenesis of IBD. Nearly every subset of T cells, including Th1, Th2, Th17, and regulatory T cells (Tregs), has been reported to play a role in the pathogenesis of IBD^4–8^. In contrast, the role of B cell lineage in the pathogenesis of IBD has been overlooked for a long time and has only received attention since the past 5 years^9–11^. As another key subset of immune cells, what roles B cells play in the pathogenesis of IBD mainly remains unclear. Many B lymphocytes terminally differentiate into plasma cells characterized by the production of various antibodies. These antibodies are strongly involved in humoral immunity and may contribute to allergy and multiple autoimmune disorders. Excessive plasmacytic infiltration in the intestinal lamina propria is a remarkable pathological characteristic of IBD^12^, suggesting a potential pathogenetic involvement of B cell lineage in IBD. IgG4, which accounts for the smallest proportion of all IgG isotypes, used to be commonly ignored because it was regarded as a noninflammatory antibody. However, its role in various autoimmune disorders, and even in cancer, has become a research focus along with the identification and prevalence of IgG4-related disease (IgG4-RD)^13,14^.

IgG4-RD is a multiorgan inflammatory condition primarily characterized by an excessive infiltration of IgG4-positive plasma cells in the affected tissues, elevated serum IgG4 concentrations, storiform fibrosis, and obliterative phlebitis, mainly affecting the pancreas, biliary tract, liver, and the retroperitoneum of the digestive tract^15^. Sporadic studies have shown high levels of mucosal and serum IgG4 in some patients with IBD. Shen *et al*.^16^ at the Cleveland Clinic investigated for the first time the characteristics of serum IgG4 in patients with IBD. In 2011, they reported one case of IgG4-associated pouchitis in a patient with UC^16^. Subsequently, this team found that elevated serum IgG4 level was associated with chronic antibiotic-refractory pouchitis among patients with IBD^17^. Rebours *et al*.^18^ compared the infiltration of mucosal IgG4+ plasma cells in intestinal tissues of patients with IBD and patients with autoimmune pancreatitis (AIP). This study demonstrated that patients with IBD had higher intestinal mucosal IgG4 counts than patients with AIP. Another study by Raina *et al*.^19^ focusing on mucosal IgG4 in IBD found that infiltration of mucosal IgG4+ plasma cells was associated with primary sclerosing cholangitis (PSC) and that mucosal IgG4 counts in patients with active UC were higher than those in patients with inactive UC. The above studies demonstrated that a rise in the level of either mucosal or serum IgG4 can be found in some sporadic patients with IBD, but there is a lack of matched case–control cohort studies comparing intestinal and serum IgG4 levels between patients with IBD and healthy individuals. To date, it has been well acknowledged that IgG4 is a critical marker for the diagnosis of IgG4-RD. In contrast‚ the role that the tissue IgG4+/IgG+ ratio of plasmacytic infiltration and the serum IgG4/IgG ratio play in diagnosing IgG4-RD remains controversial^20–22^. Besides IgG4, the characteristics of the serum IgG4/IgG ratio in IBD are also obscure and need to be identified.

Based on the above evidence, we hypothesized that IBD patients with significant level of serum and/or mucosal IgG4 may possess distinct clinical characteristics and that high level of IgG4 is a biomarker for a new subtype of IBD. Therefore, we conducted a prospective case–control cohort study characterizing serum IgG4 levels, IgG4/IgG ratios, and infiltration of mucosal IgG4+ plasma cells in Chinese patients with IBD and investigated their associations with clinical characteristics. In this study, we identified a small subset of patients with IBD exhibiting high levels of serum and mucosal IgG4, and these patients had their own distinctive clinical features. All the evidence suggests that high level of IgG4 may be a biomarker for a new subtype of IBD.

## Methods

### Study population and samples

**Figure 1:**
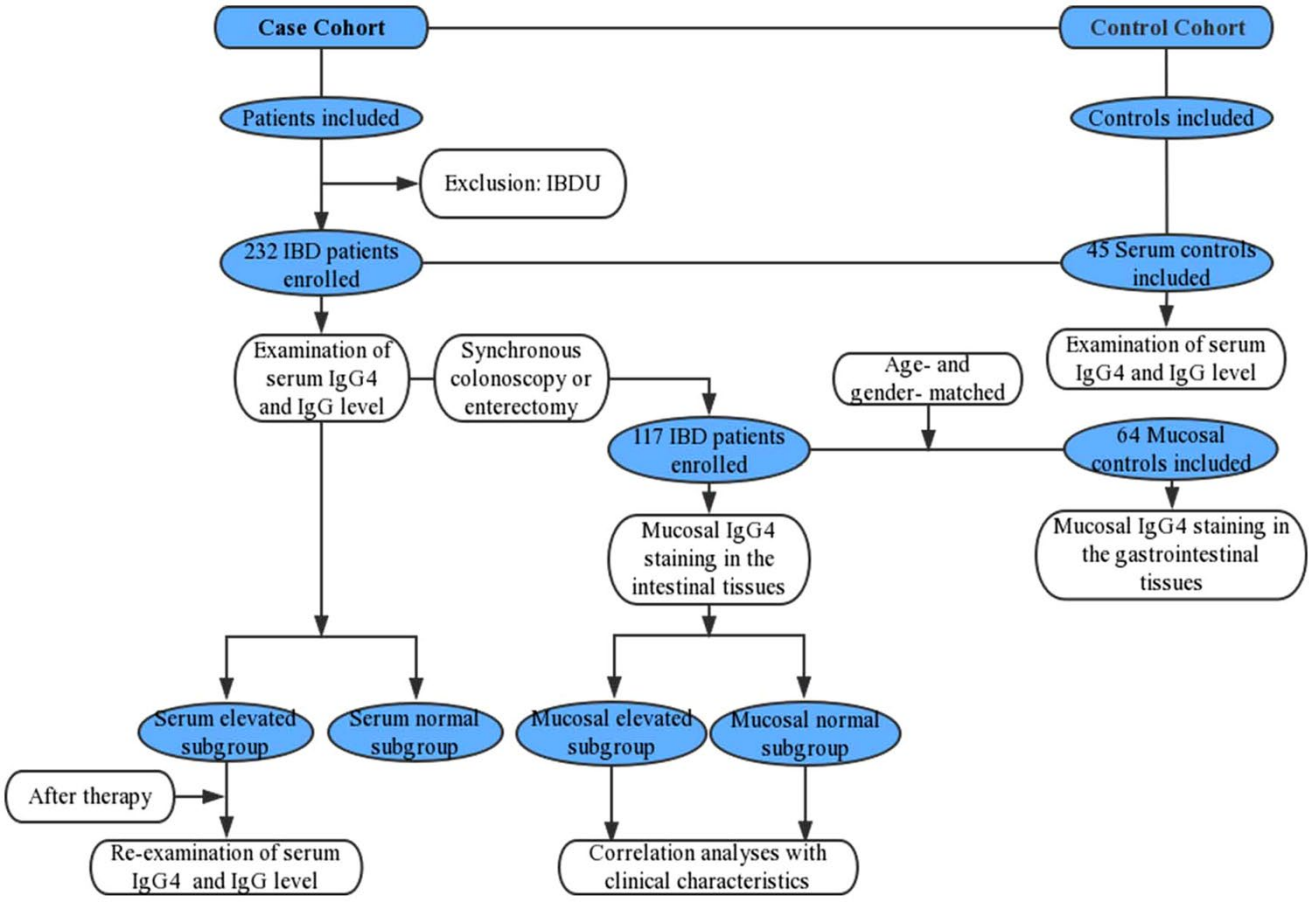
Flow chart of patient selection in the study and main study procedures.

Patients diagnosed with IBD regardless of treatment history at the West China Hospital between March 2015 and September 2017 were prospectively enrolled in this study. The confirmed diagnosis of IBD was based on accepted criteria^23^, including characteristic clinical, endoscopic, radiographic, and histological findings. Patients with IBD that coexisted with other autoimmune diseases were also included. Patients who could not be differentiated between UC and CD (IBD unclassified) were excluded. Finally, the study included 232 patients with IBD (104 UC and 128 CD). A 5-ml sample of peripheral blood was collected from each enrolled patient for measurement of serum IgG and IgG4 concentrations. A second 5-ml sample was collected from patients with high levels of serum IgG4 for re-examination of serum IgG and IgG4 concentrations after various therapies. The serum IgG4 levels and IgG4/IgG ratios in patients with IBD were compared with those in 45 healthy control individuals (age, 15–86 years). Among the 232 patients with IBD whose serum IgG4 levels were measured, biopsies of intestinal tissue during synchronous colonoscopy or enterectomy were obtained from 117 patients (58 UC and 59 CD) to assess the infiltration of mucosal IgG4+ plasma cells. The infiltration of IgG4+ plasma cells in patients with IBD was compared with that in 64 healthy control individuals (age, 15–85 years) who underwent endoscopy with normal endoscopic and pathologic presentations. The healthy controls were age- and sex- matched to patients with IBD. Patient selection and the main study procedures are listed in the flow chart (Fig. 1).

### Ethical approval

Informed consent was obtained from all patients, in accordance with the Declaration of Helsinki. This study was approved by the local Ethics Committee of West China Hospital, and all experiments in this research were performed in accordance with the relevant guidelines and regulations.

### Demographic and clinical variables

Demographic data, including age and sex, were recorded for all enrolled patients and healthy controls. Clinical data from patients with IBD, including disease phenotype, disease severity, therapies, history of intestinal surgery (mainly enterectomy, stricturoplasty, operation for perianal disease, fistula, and perforation) and coexistence of other digestive autoimmune diseases (such as PSC and AIP) were also recorded. Inflammatory markers, including C-reactive protein (CRP: cutoff level at 5 mg/L) and erythrocyte sedimentation rate (ESR: cutoff level at 26 mm/h), were measured, and disease activity indexes were evaluated following the Mayo Index for UC and the Clinical Disease Activity Index for CD.

### Examination of serum IgG and IgG4

Serum was extracted from fresh peripheral blood after centrifugation. Thereafter, serum IgG and IgG4 levels were assessed by an automatic immunology analyzer (Beckman Image 800, CA, USA) following the method of scatter turbidimetry. The cutoff levels, which were set as the upper limits of the reference ranges for these markers in our institutional laboratory, were 1.50 g/L for serum IgG4 and 15.5 g/L for serum IgG. Furthermore, patients with high levels of serum IgG4 were longitudinally followed up, and serum IgG4 and IgG levels were re-examined after therapy.

### Evaluation of histological indicators

All biopsy specimens from endoscopy or enterectomy were formalin-fixed and paraffin-embedded. The hematoxylin-eosin–stained slides of gastrointestinal biopsy specimens were evaluated for histopathologic indicators of disease severity by two independent pathologists. As previously described, active inflammation was graded as mild (cryptitis), moderate (crypt abscesses), and severe (erosions or ulcerations)^24^.

### Examination of tissue IgG4

Immunohistochemistry for IgG4+ plasma cells was performed on formalin-fixed, paraffin-embedded tissue sections. The slides were immune labeled with monoclonal antibody against IgG4 to assess the infiltration of mucosal IgG4+ plasma cells (cytoplasmic staining) using a standard principle. In brief, specimens were fixed with 10% formalin and embedded in paraffin. For IgG4 staining, deparaffinized sections were rehydrated and incubated with protein block. Thereafter, sections were incubated with monoclonal rabbit antihuman IgG4 (Abcam, Cambridge, UK) at 4 °C overnight. After washing with phosphate-buffered saline (PBS), they were treated with peroxidase-conjugated goat antirabbit polyclonal antibodies (Jinqiao, Nanjing, China) for 30 min at room temperature, developed with 3,3-diaminobenzidine and H_2_O_2_ in PBS for 3 min, and counterstained with hematoxylin. Then two independent researchers quantified and recorded the numbers of infiltrated IgG4+ plasma cells under a high-resolution microscope. The presence of >10 IgG4 cells per high-power field (HPF; ×40 field) was considered significant, as previously described^15,24^.

### Statistical analysis

Data were presented as the mean ± standard deviation (SD) or standard error (SE) for continuous variables and as counts or proportions for categorical variables. The statistical association between categorical and continuous variables was analyzed by independent samples t-test or Mann–Whitney test (comparing two classes) or by one-way analysis of variance (ANOVA) or Kruskal–Wallis test (comparing three or more classes), while the association between two categorical variables was analyzed by the χ2 test or Fisher’s exact test, as appropriate. Spearman correlation coefficient (r) was used to determine the correlation between two continuous variables. The statistical analyses were conducted using the SPSS version 20.0 (SPSS; Chicago, USA). All statistical tests were two sided. P-values < 0.05 were considered to indicate statistical significance.

### Data availability

The datasets generated and/or analyzed during the current study are available from the corresponding author on reasonable request.

## Results

### Characteristics of serum IgG4 in patients with IBD

#### General characteristics of serum IgG4 levels and IgG4/IgG ratios between patients with IBD and healthy individuals

**Figure 2:**
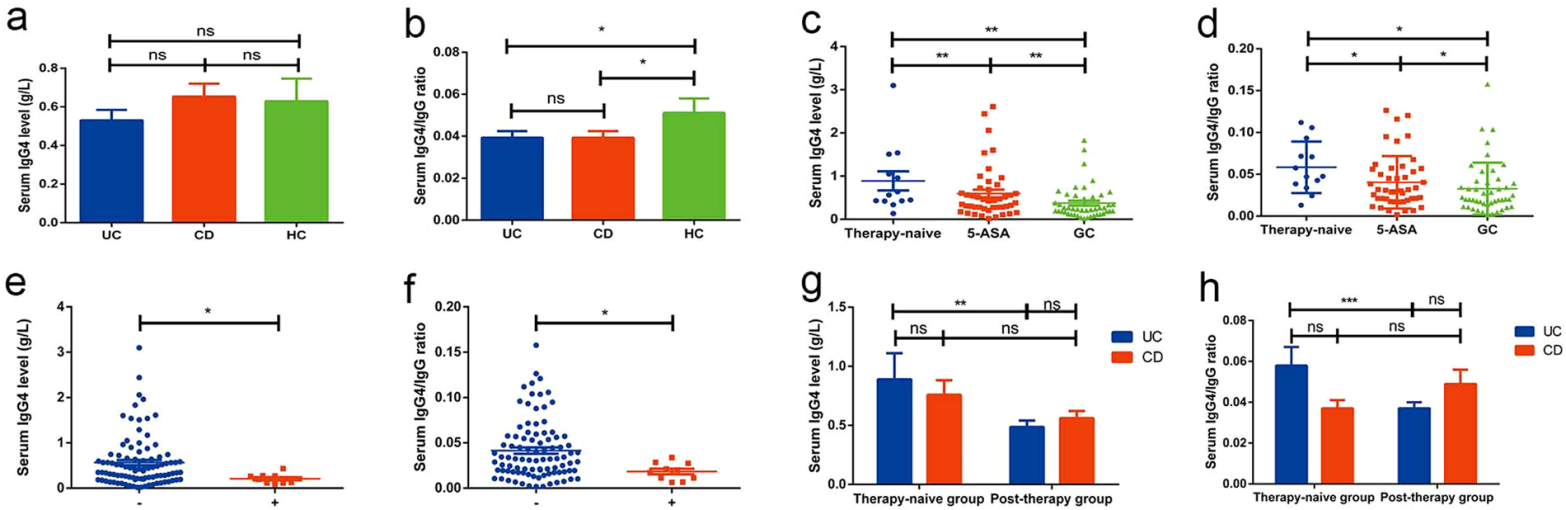
Characteristics of serum IgG4 levels in patients with IBD (104 UC & 128 CD) and 45 healthy controls. Serum IgG4 level (**a**) and IgG4/IgG ratio (**b**) among patients with UC, CD and healthy individuals; correlation analyses in patients with UC: serum IgG4 level (**c**) and IgG4/IgG ratio (**d**) were correlated with therapy; serum IgG4 level (**e**) and IgG4/IgG ratio (**f**) were correlated with history of intestinal surgery (+: patients with a history of intestinal surgery; −: patients without history of intestinal surgery); serum IgG4 level (**g**) and IgG4/IgG ratio (**h**) in therapy-naïve patients and post-therapy patients with IBD. (*p < 0.05, **p < 0.01, ***p < 0.001 and ns: not significant).

The demographic profiles of all enrolled patients with IBD and the control cohort are shown in Table 1. An overview of the clinical characteristics of all patients with IBD with respect to disease phenotype and history of intestinal surgery is also provided in Table 1. Three UC patients had coexisted with PSC, and none of patients with IBD had coexisted with AIP. The levels of serum IgG4 varied considerably in patients with IBD (range, 0.018–3.10 g/L in patients with UC and 0.004–5.25 g/L in patients with CD). The serum IgG4 levels did not differ significantly among patients with UC, patients with CD, and healthy controls (*P* > 0.05). The IgG4/IgG ratios did not differ significantly between patients with UC and CD (*P* > 0.05). The serum IgG4/IgG ratios in patients with both UC and CD were lower than in healthy controls (*P* < 0.05; Fig. 2a,b).

**Table 1:** Demographic and clinical profiles of 232 patients with inflammatory bowel disease and 45 healthy controls examined for serum IgG4.

	Ulcerative colitis	Crohn’s disease	Healthy controls
**Sex**			
Male	46	84	23
Female	58	44	22
age (years, mean ± SE)	42.88 ± 14.63	32.91 ± 14.45	56.53 ± 16.98
**Disease activity**			
Remission	0	17	
Mild	15	32	
Moderate	62	77	
Severe	27	2	
**Distribution**			
E1	4		
E2	35		
E3	65		
**Behavior**			
B1		63	
B2		30	
B3		27	
B2 + B3		8	
**Location**			
L1		17	
L2		25	
L3		81	
L1 + L4		2	
L2 + L4		1	
L3 + L4		2	
Perianal disease		39	
History of intestinal surgery	20	51	

E1, proctitis; E2, left sided; E3, extensive; B1, nonstricturing and nonpenetrating; B2, stricturing; B3, penetrating; L1, ileum; L2, colon; L3, ileocolon; L4, upper GI location.

#### Correlation analyses between serum IgG4 levels, IgG4/IgG ratios and clinical characteristics in patients with IBD

Correlation analyses showed that both serum IgG4 levels and IgG4/IgG ratios were associated with history of intestinal surgery and medical therapies in patients with UC (*P* < 0.05). To be specific, the serum IgG4 levels and IgG4/IgG ratios in therapy-naïve patients with UC were higher than those in their post-therapy counterparts, and patients with UC taking 5-aminosalicylic acid (5-ASA) had higher serum IgG4 levels and IgG4/IgG ratios than patients taking glucocorticoids (GCs; Fig. 2c,d). Patients with UC who had a history of intestinal surgery had lower serum IgG4 levels and IgG4/IgG ratios than those without a history of intestinal surgery (Fig. 2e,f). However, such an association was absent in patients with CD. Serum IgG4 levels and IgG4/IgG ratios were also not correlated with demographics (age and sex), disease phenotypes (disease extent, location, and severity), inflammatory markers (CRP and ESR), and severity indexes in patients with UC or CD.

#### Clinical characteristics of patients with IBD with high levels of serum IgG4

**Figure 3:**
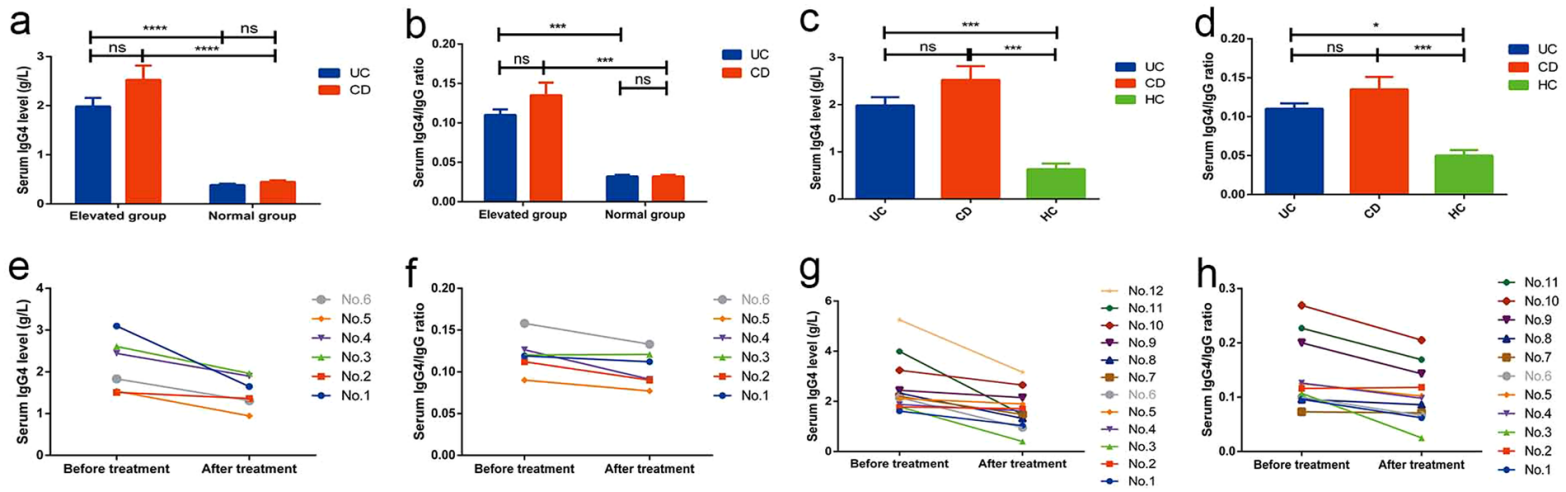
Characteristics of serum IgG4 in IBD patients (10 UC & 13 CD) with high levels of serum IgG4. Serum IgG4 level (**a**) and IgG4/IgG ratio (**b**) in patients with UC and CD in serum elevated and normal subgroups; serum IgG4 level (**c**) and IgG4/IgG ratio (**d**) in patients with UC and CD in serum elevated subgroup and healthy individuals; changes in serum IgG4 level and IgG4/IgG ratio after treatment in patients with UC (**e** and **f**) and CD (**g** and **h**) with high levels of serum IgG4. (*p < 0.05, **p < 0.01, ***p < 0.001 and ns: not significant).

Of the 232 patients with IBD in this study, 23 (9.9%) had elevated serum IgG4 (the serum elevated subgroup), as compared with 209 (90.1%) patients with normal serum IgG4 (the serum normal subgroup). Generally, serum IgG4 levels in the serum elevated subgroup were 5.18 times and 5.60 times higher than those in the serum normal subgroup for UC and CD, respectively (Fig. 3a, *p* < 0.05). Serum IgG4/IgG ratios were 1.50 times and 1.39 times higher in serum elevated subgroup than in serum normal subgroup for UC and CD respectively (Fig. 3b, *p* < 0.05). Of course, both serum IgG4 and IgG4/IgG4 ratios in serum elevated group were higher in IBD regardless of UC or CD than in normal controls (Fig. 3c,d; *p* < 0.05). There was no significant difference in serum IgG4 levels and IgG4/IgG ratios between patients with UC and CD in the serum elevated subgroup. One patient with UC in the serum elevated subgroup and two patients with UC in the serum normal subgroup had concurrent PSC.

Ten of 104 patients with UC had high levels of serum IgG4; nine of these 10 patients (90%) not only had extensive lesions (E3) but also presented with moderate-to-severe inflammation, suggesting that this subset of patients with UC was more likely to have extensive and severe lesions (Table 2). The serum IgG4 levels and IgG4/IgG ratios were not correlated with the Mayo Index, but patients with moderate-to-severe UC had higher serum IgG4 levels.

**Table 2:** Clinical characteristics of ulcerative colitis patients with high levels of serum IgG4.

Patients	Sex	Serum IgG4 (g/L)	Serum IgG (g/L)	Distribution	Disease severity	Treatment	SILBNRAT
1	M	1.51	13.5	E1	Mild	5-ASA	Y
2	F	1.54	16.5	E3	Moderate	5-ASA	NA
3	F	1.54	17.2	E3	Moderate	5-ASA	Y
4	M	2.06	21.7	E3	Moderate	5-ASA	NA
5	F	2.61	21.7	E3	Moderate	5-ASA	N
6	F	2.44	19.3	E3	Moderate	5-ASA	N
7	M	1.61	18.3	E3	Moderate	GC	NA
8	M	3.1	29.3	E3	Severe	GC	N
9	M	1.83	11.6	E3	Severe	GC	Y
10	M	1.6	13.8	E3	Severe	GC	NA

SILBNRAT: Serum IgG4 level back to normal range after treatment; M, male; F, female; E1, proctitis; E2, left sided; E3, extensive; 5-ASA, 5-aminosalicylic acid; GC, glucocorticoid; Y, yes; N, no; NA, not available.

Thirteen of 128 patients with CD had high levels of serum IgG4, they mainly exhibited ileocolonic (69.2%), inflammatory (61.5%), and moderately active (84.6%) lesions. Like patients with UC with elevated serum IgG4, this subset of patients with CD may also have relatively extensive lesions (Table 3). Surprisingly, the two patients with mild CD had slightly higher levels of serum IgG4 than those with moderate CD. This may be due to a bias caused by a small cohort of patients with mild CD or difference in the pathogenesis of UC and CD.

**Table 3:** Clinical characteristics of patients with Crohn’s disease with high levels of serum IgG4.

Patients	Sex	Serum IgG4 (g/L)	Serum IgG (g/L)	B	L	P	Disease severity	Treatment	SILBNRAT
1	F	1.61	16.7	B1	L3	Y	Moderate	GC	Y
2	M	1.78	15.4	B2	L2	Y	Moderate	AZA	N
3	F	1.79	16.8	B3	L3	Y	Moderate	AZA	Y
4	M	1.88	14.9	B1	L1	N	Moderate	AZA	N
5	F	2.05	17.6	B2	L3	N	Moderate	IFX	NA
6	F	2.12	17	B1	L3	Y	Moderate	GC	N
7	M	2.15	21.4	B1	L3	Y	Moderate	AZA	Y
8	F	2.2	30.2	B2 + B3	L3	N	Moderate	AZA	Y
9	M	2.33	21.7	B1	L1 + L4	N	Moderate	GC	Y
10	M	2.44	25.5	B1	L3	Y	Moderate	AZA	N
11	M	3.24	14.3	B3	L3	Y	Moderate	AZA	N
12	F	3.99	20	B1	L3	N	Mild	GC	Y
13	F	5.25	19.5	B3	L1 + L4	N	Mild	GC	N

SILBNRAT: Serum IgG4 level back to normal range after treatment; M: Male; F: Female; B: Behavior; B1, nonstricturing & nonpenetrating; B2, stricturing; B3, penetrating; L: Location; L1, ileum; L2, colon; L3, ileocolon; L4, upper GI location; P: Perianal disease; GC: glucocorticoid; AZA: Azathioprine; IFX: Infliximab; Y: Yes; N: No; NA: Not available.

#### Serum IgG4 in therapy-naïve patients with IBD and healthy individuals

The above results indicate that the use of medications may affect serum IgG4 levels and IgG4/IgG ratios, particularly in patients with UC. This is in line with reports that the IgG4 level may mildly decline after immunosuppressive therapy^15,24^. We further characterized serum IgG4 in the therapy-naïve subgroup of patients with IBD. In our study, 78 patients with IBD were not placed on any treatment before enrollment. In this subgroup, the number of patients with UC was much smaller than that of patients with CD, because most patients with UC had been treated in other hospitals before being referred to our hospital. This subgroup contained 13 patients with UC (10 males and three females) and 61 patients with CD (39 males and 22 females). In general, similar to the findings in patients with IBD regardless of therapy, no significant difference among therapy-naïve patients with UC, therapy-naïve patients with CD, and healthy controls was found in both serum IgG4 levels and IgG4/IgG ratios (*p* > 0.05), although serum IgG4 levels and IgG4/IgG ratios were significantly higher in therapy-naïve patients with UC than in their post-therapy counterparts described above (Fig. 2g,h). Serum IgG4 levels and IgG4/IgG ratios were not correlated with demographic and clinical profiles (history of intestinal surgery, medications, disease phenotype, inflammatory markers, and severity indexes) in therapy-naïve patients with UC or CD.

#### Follow-up of serum IgG4 levels and IgG4/IgG ratios after therapy in IBD patients with high levels of serum IgG4

In the follow-up, serum IgG4 and IgG levels were re-examined and disease activity was simultaneously re-evaluated after medical treatment in six of 10 UC patients with elevated serum IgG4. Along with decreased levels of inflammatory markers, serum IgG4 levels decreased in all six patients to various degrees (range, 0.15–1.45 g/L), and the serum IgG4/IgG ratios decreased in five patients (range, 0.005–0.035; Fig. 3e,f). Serum IgG4 levels decreased more dramatically in patients taking GC than in those taking 5-ASA.

During the follow-up, 12 of 13 CD patients with elevated serum IgG4 were re-examined for serum IgG4, and 11 were re-examined for serum IgG after treatment. The results showed that, along with decreased levels of inflammatory markers, serum IgG4 levels also decreased in all 12 patients to varying degrees (range, 0.07–2.52 g/L), and serum IgG4/IgG ratios decreased in 10 patients (range, 0.010–0.082; Fig. 3g,h). As in patients with UC in the serum elevated subgroup, serum IgG4 levels also decreased more dramatically in patients taking GC than in those taking AZA (azathioprine) in this subset of patients with CD.

### Characteristics of mucosal IgG4 in patients with IBD

#### General characteristics of infiltration of mucosal IgG4+ plasma cells between patients with IBD and healthy individuals

**Figure 4:**
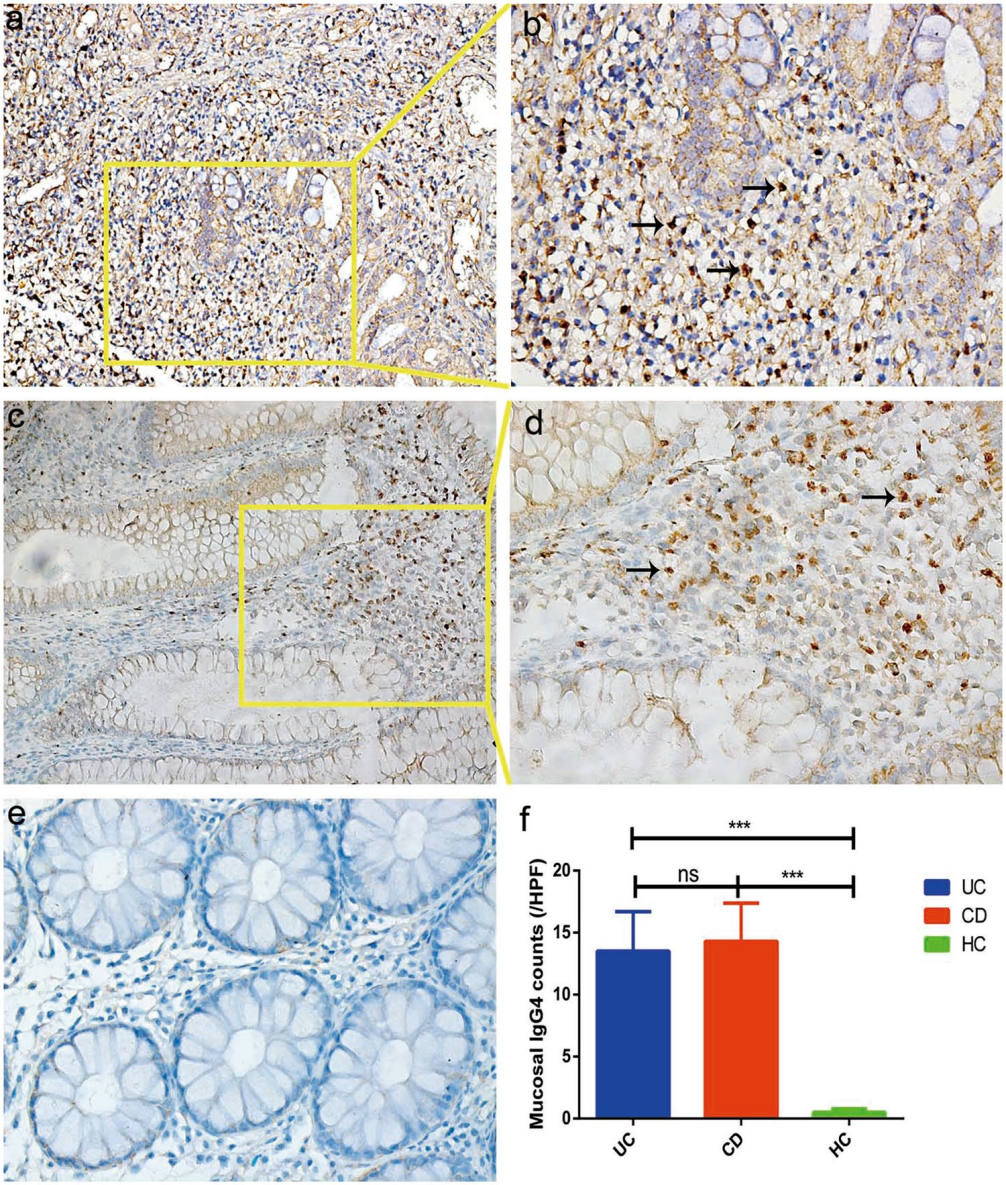
Mucosal IgG4 staining were examined in 117 patients with IBD (58 UC and 59 CD) and 64 healthy controls. Two examples of IBD patients with significant mucosal IgG4 counts: one patient with UC showing 93 IgG4+ plasma cells/HPF (**a**, x20 field; **b**, x40 field); the other patient with CD showing 61 IgG4+ plasma cells/HPF (**c**, x20 field; **d**, x40 field). Negative control of a healthy individual (**e**, x40 field). Mucosal IgG4 count was significantly higher in patients with IBD than healthy individuals (**f**). (*p < 0.05, **p < 0.01, ***p < 0.001 and ns: not significant).

In this study, 117 cases with IBD (58 UC and 59 CD) and 64 healthy controls were included to investigate the infiltration of mucosal IgG4+ plasma cells by means of immunohistochemical staining. Suppl. Table 1 shows the demographic profiles of patients with IBD and the control cohort whose mucosal IgG4 levels were measured. An overview of clinical characteristics in this group of patients with IBD with respect to disease phenotype and history of intestinal surgery is also provided in Suppl. Table 1. Generally, the IgG4+ plasma cells exhibited considerable heterogeneity among patients with IBD (range, 0–103/HPF for UC, 0–119/HPF for CD). The example for significant infiltration of IgG4+ plasma cells in one patient with UC, one with CD, and one healthy individual is shown in Fig. 4a–e. Two patients with UC that coexisted with PSC were examined with mucosal IgG4 staining; one with elevated serum IgG4 level also showed significant mucosal IgG4 counts, whereas the other one with normal serum IgG4 level had mucosal IgG4 counts <10/HPF. The mucosal IgG4 counts in patients with IBD were dramatically higher than those in the control group (*p* < 0.05; Fig. 4f). In contrast, serum IgG4 levels were comparable in patients with IBD and healthy individuals. There was no significant difference between patients with UC and those with CD in the infiltration of IgG4+ plasma cells (*p* > 0.05; Fig. 4f).

#### Correlation analyses between the infiltration of mucosal IgG4+ plasma cells and clinical characteristics in patients with IBD

**Figure 5:**
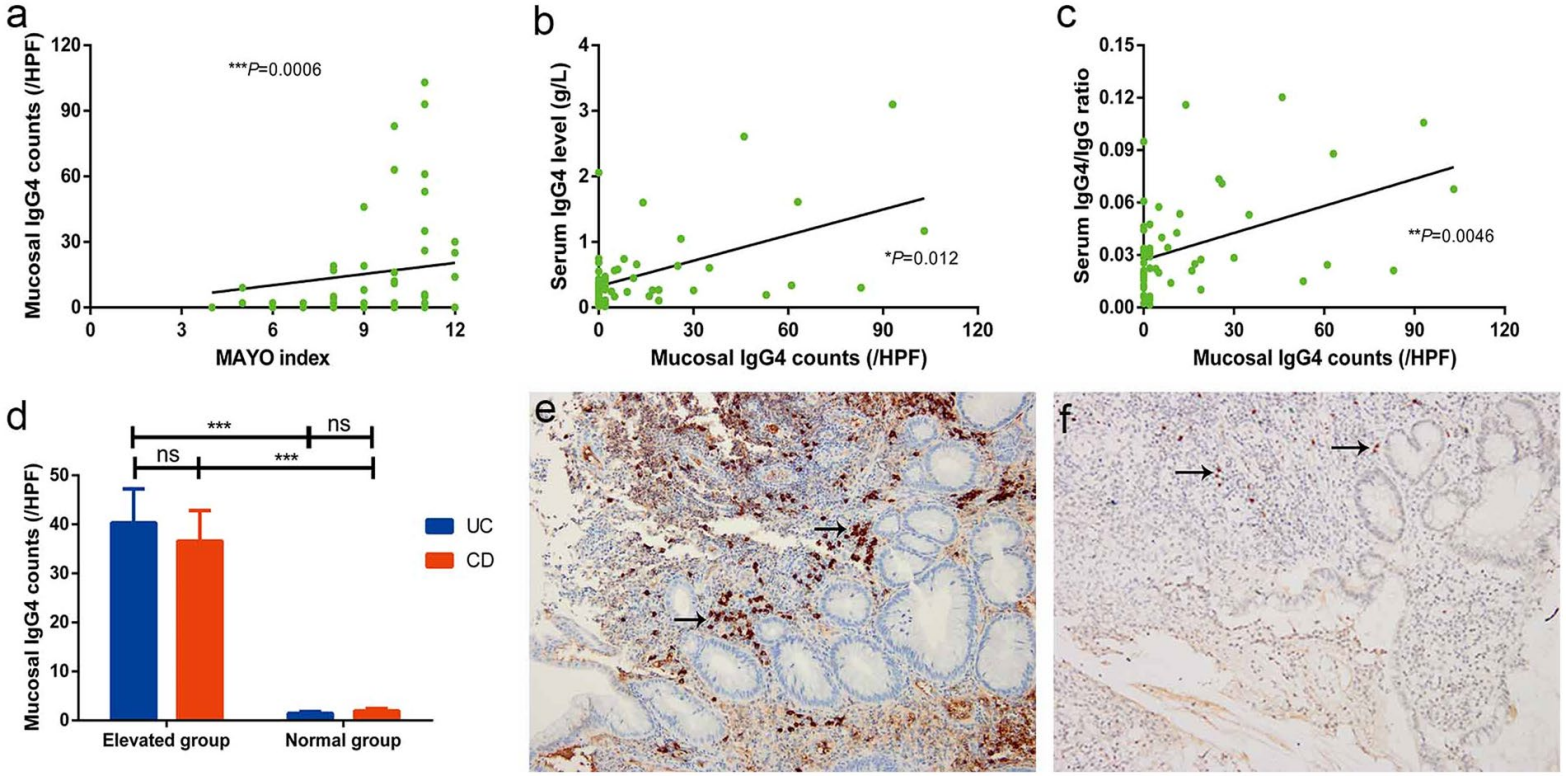
Characteristics of infiltration of mucosal IgG4+ plasma cells in patients with inflammatory bowel disease (IBD). Mucosal IgG4 count was positively associated with Mayo Index (**a**), serum IgG4 level (**b**) and IgG4/IgG ratio (**c**) in patients with ulcerative colitis (UC); (**d**) mucosal IgG4 count in patients with IBD in the mucosal elevated and normal subgroups. *p < 0.05, **p < 0.01, ***p < 0.001 and ns: not significant. Mucosal IgG4 count dramatically declined (approximately 90%) after treatment with glucocorticoid in one patient with Crohn’s disease (CD) with significant infiltration of mucosal IgG4. (**e**: before treatment and **f**: after treatment).

Correlation analyses showed that mucosal IgG4 counts were positively correlated with disease severity according to the Mayo Index in patients with UC (Fig. 5a; *p* = 0.0006). Moreover, mucosal IgG4 counts were positively associated with serum IgG4 levels and IgG4/IgG ratios in patients with UC (*p* = *0.012*, *p* = *0.0046, respectively*; Fig. 5b,c), demonstrating that increased numbers of mucosal IgG4 cells in patients with UC are associated with increased levels of serum IgG4 and vice versa. All patients with UC with high serum IgG4 levels (>1.5 g/L) had high mucosal IgG4 counts (>10/HPF) although not all patients with high mucosal IgG4 counts had high levels of serum IgG4. However, this association was not seen in patients with CD. Mucosal IgG4 counts were also not correlated with other demographic and clinical features (disease phenotype, therapy, history of intestinal surgery, and CRP and ESR inflammatory markers) in patients with UC or CD.

#### Clinical characteristics of patients with IBD with significant mucosal IgG4 counts

Among the 117 cases who underwent immunohistochemical staining, significant infiltration of mucosal IgG4+ plasma cells (defined as >10 cells per HPF) was seen in 39 patients with IBD (33.3%). In contrast, as discussed above, 23 in 232 patients with IBD (9.9%) demonstrated increased levels of serum IgG4 (>1.5 g/L). Generally, infiltration of mucosal IgG4+ plasma cells in the mucosal elevated subgroup was 28 times higher than that in the mucosal normal subgroup among UC patients (*p* < 0.05; Fig. 5d), and it also is 19 times higher in CD (*p* < 0.05; Fig. 5d). There was no significant difference in infiltration of mucosal IgG4+ plasma cells between patients with UC and CD in the mucosal elevated subgroup.

Of the 18 patients with UC with high levels of mucosal IgG4, 77.8% had extensive lesions (E3), and 88.9% showed moderate-to-severe inflammation under microscopy. Twenty-one patients with CD had high levels of mucosal IgG4; they mainly exhibited ileocolonic (76.2%), inflammatory (57.1%), and moderately active (66.7%) lesions. As in IBD patients with high levels of serum IgG4, we can speculate that this subset of patients with IBD with high levels of mucosal IgG4 may be at risk of extensive and severe lesions than others.

#### Characteristics of the consistency of high levels of serum and mucosal IgG4 in patients with IBD

Among the 117 patients with IBD, eleven patients (9.4%) with IBD (four with UC and seven with CD) consistently exhibited high levels of serum and mucosal IgG4. Among the 39 patients with elevated mucosal count, 28.2% of patients with IBD with significant mucosal IgG4 counts (>10/HPF) also had high levels of serum IgG4 (>1.5 g/L); in contrast, all 23 patients with IBD with high levels of serum IgG4 had significant mucosal IgG4 counts. Two patients with CD were re-examined by colonoscopy after treatment with GC. Immunohistochemical IgG4 staining found that the infiltration of mucosal IgG4 cells in both patients was dramatically decreased (Fig. 5e,f).

## Discussion

This prospective case–control study demonstrated that a small subset of patients with IBD can be characterized by high levels of serum and mucosal IgG4. These patients are generally prone to more severe and extensive lesions. The levels of serum and mucosal IgG4 decreased after treatment with GCs or other immunosuppressants. These characteristics indicate that high level of IgG4 may be a biomarker for a new subtype of IBD.

Patients with IBD and healthy individuals had comparable levels of serum IgG4, whereas patients with IBD had dramatically higher mucosal IgG4 counts than healthy individuals. Among patients with IBD, not all patients with significant infiltration of mucosal IgG4 cells (>10/HPF) had serum IgG4 levels >1.5 g/L. For example, immunohistochemical study found that 39 in 117 cases (33.3%) exhibited increased numbers of mucosal IgG4 cells; in contrast, serum measurement revealed that only 23 in 232 patients (9.9%) with IBD had elevated serum IgG4 levels. However, in patients with UC, mucosal IgG4 counts were positively correlated with serum IgG4 levels although quite a number of patients with significant infiltration of IgG4 cells had serum IgG4 concentrations <1.5 g/L. These differences may result from an excessive infiltration of plasma cells, particularly IgG4-expressing plasma cells, in the inflamed intestinal mucosa in a subset of patients with IBD. Another issue to be noted is that the upper limit of normal (ULN) of serum IgG4 in our study (1.5 g/L) was higher than the values defined in other studies (range, 1.12–1.40 g/L) due to different laboratory methods^17,19,25^. Most previous studies focused on the infiltration of mucosal IgG4 cells in IBD, and only a small number of patients were examined for serum IgG4. One of the largest studies exploring serum IgG4 in patients with IBD was conducted by Navaneethan *et al*.^17^, who reported that approximately 8% of patients with IBD presenting with pouchitis had elevated serum IgG4. This study showed that these patients were more likely to have an antibiotic-refractory phenotype. With a similar elevated ratio of serum IgG4 (9.9%) in patients with IBD in our study, we found that patients with IBD with elevated serum or mucosal IgG4 were more likely to have extensive and severe lesions. Moreover, we surprisingly found that the serum IgG4/IgG ratios in patients with IBD were lower than those in healthy individuals, in contrast to significantly higher serum IgG4/IgG ratios in IBD patients with higher serum and mucosal IgG4 levels compared to those in healthy individuals. Sporadic studies have indicated the diagnostic value of tissue IgG4+/IgG+ plasma cells and serum IgG4/IgG ratios in IgG4-RD. Lehman *et al*.^22^ at the Mayo Clinic indicated that a moderately increased tissue IgG4/IgG ratio was not specific for cutaneous IgG4-RD, while another meta-analysis by Deng *et al*.^20^ proposed a modest effect of the tissue IgG4/IgG ratio of plasmacytic infiltration in diagnosing IgG4-RD. Song *et al*.^21^ also found that compared with IgG4 alone, combined measurement of serum IgG4 and IgG could increase the diagnostic sensitivity of AIP. Therefore, we speculated that a small subset of patients with IBD, particularly those who are characterized by high levels of serum and mucosal IgG4, may show a predominant proportion of serum/mucosal IgG4 among all IgG isotypes, underlying a monoclonal/polyclonal expansion of IgG4-expressing B cells via an unknown pathway.

We also found that IgG4 serum levels, IgG4/IgG ratios, and mucosal IgG4 infiltration were comparable in patients with UC and CD regardless of therapy. In contrast to our results, a study by Virk *et al*.^24^ at the Harvard Medical School reported that the rate of significant infiltration of mucosal IgG4 count (>10/HPF) in patients with UC was 38%, compared with 5% in patients with CD. Mucosal IgG4 counts were significantly higher in patients with UC than in patients with CD. Virk’s study focused on a cohort of therapy-naïve patients with IBD, whereas quite a number of patients in our study who were examined with mucosal IgG4 staining had received various therapies before enrollment, particularly patients with UC. However, differences in composition between the two studies cannot explain the divergent results. It remains unclear whether there exists an ethnic difference in the distribution of mucosal IgG4-expressing plasma cells between Chinese and Western patients with IBD, underlying a difference in genetic pathogenesis. Despite similar levels of serum and mucosal IgG4 in patients with UC and CD, it was detectable in our study that patients with UC possess some distinct characteristics, such as an association between serum IgG4 levels and a history of intestinal surgery or medication use, a positive correlation between mucosal IgG4 cell counts and serum IgG4 levels, IgG4/IgG ratios, and Mayo Index. All such characteristics were absent in subjects with CD.

The pathogenesis of IBD is complex, with multiple factors involved. A strong consensus has indicated that there are substantial differences between the immune mechanisms of the two IBD subsets. For example, CD is characterized by increased production of the Th1 cytokines interleukin-12 (IL-12) and interferon γ, whereas UC is characterized by increased production of the Th2 cytokines IL-5 and IL-3. Furthermore, accumulating evidence suggests that intricate etiological differences also exist among different individuals with either UC or CD. For example, Alonso *et al*.^26^ identified four risk loci associated with various clinical phenotypes of CD. The complicated pathogenesis of IBD may partially account for current problems in treatment. To date, therapy for IBD mainly consists of 5-ASA, GC, immunosuppressants, and biological agents. Although biological agents, such as the widely used infliximab, are considered among the most promising medications, they cannot be of benefit for all patients with IBD because of problems of primary or secondary loss of response. This strongly indicates that further classification of IBD based on differences in genetic and immune pathogenesis is warranted for individual decision making and switching therapy for a given patient with IBD. The roles of IgG4-expressing plasma cells in the pathogenesis of this subset of IBD are worthy of further research, and the related signaling may contribute to personalized therapy.

So far, it still remains unknown about whether and how IgG4-expressing B cells directly function in the pathogenesis of various inflammatory autoimmune diseases. It is speculated that these IgG4+ B cells play either a pathogenic or a protective role, or that they are just secondary bystanders in this subset of IBD. What we can know, however, is that high levels of mucosal and serum IgG4 are detected in a small subset of patients with IBD who are more likely to have extensive lesions, particularly patients with UC. Furthermore, the level of serum IgG4 or tissue infiltration of IgG4+ plasma cells could decrease along with the decline in disease activity after treatment. The above observations imply that IgG4+ B cells may be involved in the immune response in this subset of IBD via an unknown pathway. Although there has been a lack of studies exploring the role of IgG4+ B cells in the pathogenesis of IBD, and the relationship between IBD and IgG4-RD is still obscure, published findings, such as the following, on the pathogenesis of IgG4-RD and IBD may suggest a potential role of IgG4 in IBD. As the most abundant inflammatory cells in the lymphocytic infiltration in IgG4-RD lesions, T cells were considered to drive the pathogenesis of the disease in IgG4-RD^13,15,27^. Early studies identified excessive Th1- and Th2-type repertoires in the lesions of IgG4-RD, giving rise to a debate on whether Th1 or Th2 immune responses are involved in the pathogenesis of IgG4-RD^28–30^. This argument was broken by a series of studies conducted by Stone *et al*. at the Harvard Medical School. Their team first demonstrated that only patients with a history of allergy could exhibit expanded Th2-type repertoires, and more importantly, clonal expansion of CD4+ cytotoxic lymphocytes (CTLs) were found to be involved in the pathogenesis of IgG4-RD^31,32^. The team further demonstrated that the pathogenesis of IgG4-RD was associated with lesional infiltration of CD4+ Granzyme A (GZMA)+ CTLs, which could secrete interferon γ, IL-1β, and transforming growth factor-β1, and the ratio of these CTLs was correlated with the numbers of involved organs^33^. Another study by this team identified an expansion of CD19+CD27+CD20−CD38hi plasmablasts, which could be depleted by anti-CD20 treatment, in IgG4-RD patients, and the re-emergence of the *de novo* distinct oligoclonal plasmablasts was correlated with relapse of IgG4-RD^34^. Therefore, it is possible that a collaboration between unidentified specific T cells and IgG4 plasma cells/plasmablasts is involved in the pathogenesis of IBD related to high levels of serum and mucosal IgG4.

For a long time, IgG4 was thought to lack pathogenic activity because of its failure to bind complement and its low affinity for Fcγ receptors (FcγR)^35^. This means that it is commonly thought as being a bystander and cannot directly play a role in the pathogenesis of IgG4-RD. However, accumulating evidence indicates that IgG4 can bind FcγRI (CD64), FcγRIIa (CD32), and FcγRIIIb (CD16)^36–39^. In 2013, Uo *et al*.^9^ at Keio University identified an expansion of CXCR4+ IgG-expressing plasma cells in the affected mucosa of patients with UC. This distinct subset of plasma cells could bind FcγRI and FcγRII, and they could further induce the activation of pathogenic intestinal CD14+ macrophages in patients with UC. As described above, IgG4 can also bind FcγRI and FcγRIIa, indicating a potential involvement of monocytes/macrophages in the pathogenesis of this subset of IBD with a high IgG4 level, inducing an IgG4-immune complex-FcγR signaling in the pathogenetic mechanisms. The antigens, whether autoantigens or microbes, that drive the expansion of specific IgG4+ B cells also need to be identified in further research.

Our study made interesting findings that a small subset of patients with IBD showed an elevated level of IgG4, and they exhibited some distinctive clinical features. This study, however, had some limitations. First, considerable numbers of patients with IBD, particularly those with UC, were on medications before their inclusion in the study. Therefore, this study could not provide a therapy-naïve overview of IgG4 characteristics for all of patients with IBD. Second, the study was conducted at a single medical center, therefore, we only detected a small number of patients with IBD who had high levels of IgG4. More prospective studies from different medical centers are warranted to further characterize this subset of patients with IBD. Third, we characterized serum and mucosal IgG4 levels in Chinese patients with IBD and identified a subset of IBD patients with high IgG4 levels. IgG4 has been widely reported as just a marker for the diagnosis of IgG4-RD in the literature. Some studies, however, recently suggest a pathogenic possibility of IgG4 in the pathogenesis of IBD. More studies focusing on the role of B cells and plasma cells in the pathogenesis of IBD are warranted.

In conclusion, this study characterized a new subset of IBD patients with high levels of serum and mucosal IgG4. These patients with IBD were more likely to have severe and extensive lesions, and the high levels of serum and mucosal IgG4 decreased after treatment with glucocorticoids or other immunosuppressants. These characteristics indicate that high level of IgG4 may be a biomarker for a new subtype of IBD. Although it remains unclear whether IgG4+ B cells play a pathogenic role in this subset of IBD, it is important to explore the related collaboration between IgG4+ B cells and other immune cells, such as T cells, monocytes, and others. Elucidation of the mechanism can contribute to personalized therapy for this subset of patients with IBD.

## Electronic supplementary material

Dataset 1
