# Supplementary material for: High level of IgG4 as a biomarker for a new subset of inflammatory bowel disease
Source: Sci Rep. 2018 Jul 3;8:10018. doi: 10.1038/s41598-018-28397-8 (PMC6030091; doi:10.1038/s41598-018-28397-8)
Supplement: Supplementary file 1 — Dataset 1 [file 41598_2018_28397_MOESM1_ESM.docx]

**High level of IgG4 as a biomarker for a new subset of inflammatory bowel disease**

Zhujun Wang^1^, Min Zhu^1^, Chengxin Luo^1^, Yu zhen^1^, Jingxi Mu^1^, Wenyan Zhang^2^, Qin Ouyang^1^, Hu Zhang^1,^

^1^Department of Gastroenterology, West China Hospital, Sichuan University, Chengdu, Sichuan, China; ^2^Department of Pathology, West China Hospital, Sichuan University, Chengdu, Sichuan, China.

Correspondence and requests for materials should be addressed to H.Z. (email: zhanghu@scu.edu.cn)

**Keywords:** IgG4, biomarker, new subset, inflammatory bowel disease


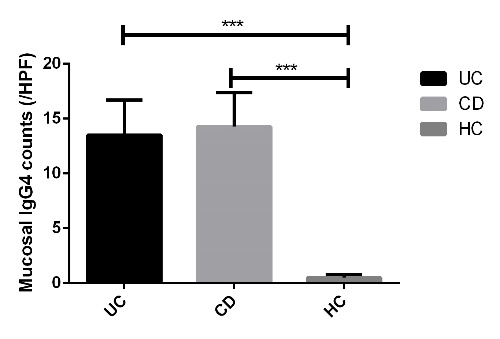
Suppl. Table 1. Demographic and clinical profiles of 117 patients with inflammatory bowel disease and 64 healthy controls examined for mucosal IgG4.

|  | Ulcerative colitis | Crohn’s disease | Healthy controls |
| --- | --- | --- | --- |
| Sex |  |  |  |
| Male | 34 | 34 | 30 |
| Female | 24 | 25 | 34 |
| Age (years, mean ± SE) | 44.60 ± 15.79 | 29.76 ± 11.77 | 49.09 ± 13.91 |
| Disease activity |  |  |  |
| Remission |  | 4 |  |
| Mild | 7 | 17 |  |
| Moderate | 35 | 38 |  |
| Severe | 16 |  |  |
| Distribution |  |  |  |
| E1 | 1 |  |  |
| E2 | 22 |  |  |
| E3 | 35 |  |  |
| Behavior |  |  |  |
| B1 |  | 30 |  |
| B2 |  | 16 |  |
| B3 |  | 8 |  |
| B2 + B3 |  | 5 |  |
| Location |  |  |  |
| L1 |  | 3 |  |
| L2 |  | 9 |  |
| L3 |  | 43 |  |
| L1 + L4 |  | 2 |  |
| L2 + L4 |  | 0 |  |
| L3 + L4 |  | 2 |  |
| Perianal disease |  | 18 |  |
| History of intestinal surgery | 7 | 24 |  |

E1, proctitis; E2, left sided; E3, extensive; B1, nonstricturing & nonpenetrating; B2, stricturing; B3, penetrating; L1, ileum; L2, colon; L3, ileocolon; L4, upper GI location.
